# An optimized MALDI MSI protocol for spatial detection of tryptic peptides in fresh frozen prostate tissue

**DOI:** 10.1002/pmic.202100223

**Published:** 2022-03-03

**Authors:** Therese S. Høiem, Maria K. Andersen, Marta Martin‐Lorenzo, Rémi Longuespée, Britt S.R. Claes, Anna Nordborg, Frédéric Dewez, Benjamin Balluff, Marco Giampà, Animesh Sharma, Lars Hagen, Ron M.A. Heeren, Tone F. Bathen, Guro F. Giskeødegård, Sebastian Krossa, May‐Britt Tessem

**Affiliations:** ^1^ Department of Circulation and Medical Imaging NTNU ‐ Norwegian University of Science and Technology Trondheim Norway; ^2^ Maastricht MultiModal Molecular Imaging Institute (M4I) Maastricht University Maastricht Netherlands; ^3^ Department of Clinical Pharmacology and Pharmacoepidemiology Heidelberg University Hospital Heidelberg Germany; ^4^ Department of Biotechnology and Nanomedicine SINTEF Industry Trondheim Norway; ^5^ Department of Clinical and Molecular Medicine NTNU ‐ Norwegian University of Science and Technology Trondheim Norway; ^6^ PROMEC Core Facility for Proteomics and Modomics NTNU ‐ Norwegian University of Science and Technology and the Central Norway Regional Health Authority Norway Trondheim Norway; ^7^ Clinic of Laboratory Medicine St. Olavs Hospital Trondheim University Hospital Trondheim Norway; ^8^ Department of radiology and nuclear medicine St. Olavs Hospital Trondheim University Hospital Trondheim Norway; ^9^ K.G. Jebsen Center for Genetic Epidemiology Department of Public Health and Nursing NTNU ‐ Norwegian University of Science and Technology Trondheim Norway; ^10^ Department of Surgery St. Olavs Hospital Trondheim University Hospital Trondheim Norway

**Keywords:** fresh frozen, MALDI mass spectrometry imaging, peptides, prostate tissue, proteomics

## Abstract

MALDI MS imaging (MSI) is a powerful analytical tool for spatial peptide detection in heterogeneous tissues. Proper sample preparation is crucial to achieve high quality, reproducible measurements. Here we developed an optimized protocol for spatially resolved proteolytic peptide detection with MALDI time‐of‐flight MSI of fresh frozen prostate tissue sections. The parameters tested included four different tissue washes, four methods of protein denaturation, four methods of trypsin digestion (different trypsin densities, sprayers, and incubation times), and five matrix deposition methods (different sprayers, settings, and matrix concentrations). Evaluation criteria were the number of detected and excluded peaks, percentage of high mass peaks, signal‐to‐noise ratio, spatial localization, and average intensities of identified peptides, all of which were integrated into a weighted quality evaluation scoring system. Based on these scores, the optimized protocol included an ice‐cold EtOH+H_2_O wash, a 5 min heating step at 95°C, tryptic digestion incubated for 17h at 37°C and CHCA matrix deposited at a final amount of 1.8 μg/mm^2^. Including a heat‐induced protein denaturation step after tissue wash is a new methodological approach that could be useful also for other tissue types. This optimized protocol for spatial peptide detection using MALDI MSI facilitates future biomarker discovery in prostate cancer and may be useful in studies of other tissue types.

**Abbreviations**
AR: Antigen retrival
CytC: Cytochrome C
FFPE: formalin‐fixed paraffin‐embedded
HES: hematoxylin, eosin and saffron
ITO: indium tin oxide
LS: localization score
MSI: mass spectrometry imaging
MQ: MaxQuant
PSM: peptide‐spectra‐matches
QE: quality evaluation

## 1 INTRODUCTION

Proteins carryout major processes in living organisms and aberrant protein regulation is a key player in disease progression [1]. Large‐scale proteomics studies are therefore important in biomarker research. Most forms of cancer are heterogeneous with tumor tissue containing different cell types and cancer cells present. Such tumor tissue contains spatial information that will be lost in most bulk proteomic methods. Therefore, mass spectrometry imaging (MSI), a technique able to spatially resolve a wide range of molecules directly in tissue, has become a rapidly growing methodological approach to analyze a range of diseases [2, 3, 4, 5, 6, 7], including prostate cancer [8, 9, 10, 11]. Matrix‐assisted laser desorption/ionization (MALDI) is the most common MSI method for spatial detection of not only proteins [2, 8, 9, 10, 12, 13] and peptides [14], but also for glycans [15, 16], metabolites [11, 17], lipids [11, 18] and recently also for metal elements [3, 4].

Prostate cancer tissue is inherently heterogeneous, and only a few studies have successfully identified differentially expressed intact protein levels using MALDI MSI comparing cancer and benign fresh frozen prostate tissue [8, 9, 10]. Proteins identified as discriminating tumor from benign prostate tissue include mitogen‐activated protein kinase/extracellular signal‐regulated kinase 2 and overexpression of biliverdin reductase B [10, 19]. In a recent study on fresh frozen prostate cancer tissue, MALDI MSI was used to detect different levels of tryptic peptides on cancerous tissues in comparison to benign tissue. The authors reported two proteins (ribonuclease T2 and a heat shock protein) as potential biomarkers for aggressive prostate cancer tissue [5]. Additionally, other studies have found differential tryptic peptides patterns in formalin‐fixed paraffin‐embedded (FFPE) prostate cancer tissue [6, 7, 20]. The optimal protocol for detection of intact proteins or peptides with MALDI MSI depends on tissue characteristics and sample type (e.g. fresh frozen or FFPE tissue), the desired mass resolution, *m/z* interval, and sensitivity of the MSI instrument.

A critical part of MALDI MSI peptide detection is the use of an efficient, robust and reproducible sample preparation procedure optimized to the target tissue investigated [21, 22]. Small deviations from an optimized sample preparation protocol can induce variations in mass intensity and spatial localization of analytes affecting the validity of the results. High‐quality tissue collection and preservation are essential to limit analyte degradation and maintain the integrity of the tissue [22, 23]. Commonly, fresh frozen tissue has been considered the most suitable material for analyses in MALDI MSI [24, 25]. Fresh frozen tissue requires careful handling and storage at low temperatures (‐80°C) to avoid protein degradation and analyte delocalization [24, 25, 26]. An alternative to fresh frozen tissue is FFPE tissue, frequently implemented in clinical pathology and available in biobanks worldwide [6, 7, 20]. FFPE tissue preserves tissue morphology and provides easy long‐term storage at room temperature (RT). However, removal of paraffin embedding and reversing the formalin‐induced crosslinks (antigen retrieval) adds handling time to the sample preparation procedure and exposes the tissue for harsher mechanical and chemical treatment [24, 27]. By using fresh frozen tissue, we limit additional tissue processing, thereby limiting technical variation in peptide detection in MSI experiments.

The main steps of proteolytic peptide detection with MALDI MSI on fresh frozen tissue samples typically include cryosectioning, washing the tissue sections to remove interfering compounds, on‐tissue enzymatic digestion, matrix deposition and MALDI MSI data acquisition. Most of these steps, if not all, need to be optimized for every tissue type analyzed. Several methodological variants of sample preparation for peptide detection with MALDI MSI have been described in literature, but there is no clear consensus on an optimal protocol for fresh frozen tissues in general [28].

The aim of this study was to establish an optimal protocol for reproducible analyses of tryptic peptides using high‐speed MALDI MSI on fresh frozen prostate tissue samples for a subsequent high quality clinical study. We performed a series of experiments, following a linear experimental design, where new tests were adjusted based on previous results to achieve an optimized working protocol. Quantity and quality of peptide detection were evaluated by several criteria: number of peaks detected on tissue, number of excluded peaks (false positives), signal delocalization due to analyte diffusion/migration, spectral quality estimation by signal‐to‐noise ratio (S/N), and signal intensities of selected peptides.

## 2 METHODS

### 2.1 Materials

All chemicals and organic solvents were purchased through Sigma‐Aldrich (Merck Life Science AS, Oslo, Norway) in the highest purity available, if not stated otherwise. Trypsin (lyophilized) was purchased from Promega (Cat No. V5111) and Sigma‐Aldrich (Cat No. T6567).

### 2.2 Tissue sample collection

Prostate tissue specimens were collected from five patients after radical prostatectomy (in the period 2007–2008) by Biobank1 at St. Olavs hospital, Trondheim University Hospital (Trondheim, Norway). As this is a protocol development study, only samples with noncancerous histology (epithelial glands and stroma) were chosen to reduce sample variability. Cylindrical samples (⌀ = 3 mm) were drilled in frozen condition from 2 mm thick whole‐mount fresh frozen prostate slices as described by Bertilsson et al. and stored at −80°C [29]. This study was approved by the Regional Committee for Medical and Health Research Ethics, Central Norway (identifier 2017/576) and was conducted according to EU regulations and ethical guidelines. Written informed consent was collected from all prostate cancer patients participating in this study.

### 2.3 Tissue sectioning

Prostate tissue samples (n = 14) were cryosectioned (‐20°C) at a thickness of either 4 μm (n = 16) or 10 μm (n = 109) and mounted onto indium tin oxide (ITO) covered glass slides (Bruker Daltonics, Bremen, Germany). Up to four sections were placed on each ITO slide (Supplementary Figure S1) and in total 125 sections were included in the study. After cryosectioning, the slides were stored at −80°C until analysis (maximum 2 months).

### 2.4 MALDI MSI Sample preparation workflow

**FIGURE 1:**
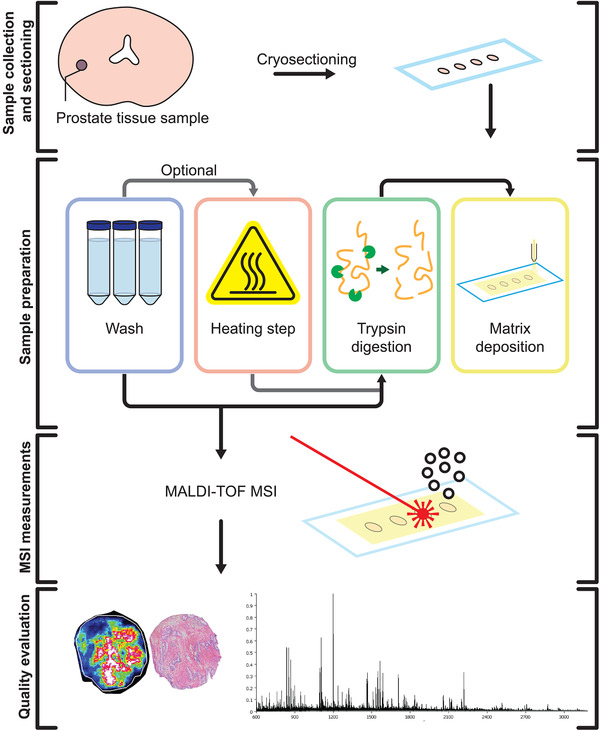
Experimental workflow of peptide analysis using MALDI MSI on fresh frozen prostate tissue samples. Sections (n = 125) from 14 samples were included in the optimization

The protocol workflow from tissue extraction and preparation until MSI analyses is presented in Figure 1. We followed a linear experimental design, where the selected protocol parameters to test were based on previous lab results and planned sequentially during the optimization period. At least three replicates were carried out per method. In total, 25 unique methods were tested and distributed accordingly.

**Statement of significance**

Mass spectrometry imaging (MSI) has become a popular set of techniques for analyzing heterogeneous cancer tissue, as it allows for spatial mapping of biological molecules, including peptides. However, identifying the optimal sample preparation protocol for peptide detection with MSI is not straightforward and require method optimalization for the target material. This study describes our journey of method optimization, where every preparation step was tested and that eventually resulted in an optimal protocol for tryptic peptide detection with improved reproducibility. The optimization step with the highest impact on signal yield and image quality was to include a heating step prior to enzymatic digestion. Although anyone starting experiments with MSI tryptic peptide detection will need to test and optimize their sample preparation protocol, we suggest that exposing the fresh frozen tissue sections to brief heating will likely improve the effect of subsequent enzymatic digestion and thereby give higher peptide signals.

### 2.5 Optimization of tissue wash

Prostate tissue sections on ITO slides were thawed at room temperature in a vacuum desiccator before washing with either Carnoy's, EtOH+H_2_O, ice‐cold EtOH+H_2_O or EtOH+short H_2_O wash. Carnoy's wash consisted of 30s steps of 70% EtOH, 100% EtOH, Carnoy's solution (60% EtOH, 30% chloroform and 10% acetic acid) and ddH_2_O with 0.2% trifluoroacetic acid (TFA), ended by 5 min of ddH_2_O. Both EtOH+H_2_O and ice‐cold EtOH+H_2_O consisted of 3 × 2 min 100% EtOH and 2 × 5 min ddH_2_O steps, while EtOH+short H_2_O included 3 × 2 min 100% EtOH and 2 × 1.5 min ddH_2_O steps. Solutions for all washes were kept and used at room temperature apart from the ice‐cold EtOH+H_2_O wash where ethanol was kept and used at −80°C and ddH_2_O at 4°C. After washing, the slides were dried in a vacuum desiccator.

### 2.6 Heating step

Various heating strategies were applied to test for potential effects on the following trypsin digestion. The heating step was performed after the washing step and four different experimental procedures were tested: 1) slides placed in a humidity chamber with a saturated K_2_SO_4_ solution for 10 min at 70°C [7]; 2) slides placed in a staining jar filled with saturated K_2_SO_4_ (covering the bottom of the jar, not in contact with the tissue) and heated at 95°C for 5 min under water‐vapor; 3) using heat‐induced antigen retrieval (AR) in a 2100 Retriever (Aptum Biologics Ltd., UK) or a kitchen pressure cooker with 10 mM citric acid (pH = 6.0) as AR buffer for 30 min at 121°C, and 4) no heating step was applied. For procedure 3), the AR buffer was rinsed off with ddH_2_O (2 × 1 min) and the slides were dried in a vacuum‐desiccator.

### 2.7 Trypsin digestion

Lyophilized trypsin was reconstituted immediately prior to usage in cold H_2_O to give a final concentration of either 0.02 or 0.1 μg/μL. To validate trypsin activity, 1 μL of Cytochrome C (CytC, 1 mg/mL) was spotted on each slide [30]. Trypsin was applied using a SunCollect sprayer (SunChrom, Friedrichsdorf, Germany) or an HTX M5 Sprayer (HTX‐Technologies, Chapel Hill, North Carolina, USA), abbreviated “HTX sprayer” throughout the paper, using customized and optimized spraying protocols for each sprayer (technical details in Supplementary Table S1). After trypsin application, the slides were incubated in a humidity chamber for 2 h or 17 h at 50°C or 37°C, respectively. To keep humidity constant, the chamber was filled with either 50% (v/v) methanol or saturated aqueous K_2_SO_4_ solution. Thereafter, trypsin digestion was terminated by matrix application. The applied trypsin density (W) was 1.3 and 6.7 ng/mm^2^ when using 0.02 and 0.1 μg/μL trypsin solutions with the HTX sprayer, and 3.3 and 16.7 ng/mm^2^ when using 0.02 and 0.1 μg/μL trypsin solutions with the SunCollect sprayer. Trypsin density was calculated according to Supplementary Equation S1.

### 2.8 Matrix solution and deposition

Three different matrix solutions of α‐Cyano‐4‐hydroxycinnamic acid (CHCA) were compared for the analysis of peptides: 5 mg/mL, 7 mg/mL or 10 mg/mL, all of which were dissolved in 50% (v/v) acetonitrile (ACN) incl. 0.2% TFA. CHCA matrix was sprayed with the two same sprayers mentioned above (SunCollect (SunChrome) and HTX TM‐Sprayers (HTX Technologies)). Collectively for all the tests performed on the HTX sprayer (HTX Technologies), N_2_ flow rate was always kept at 10 psi, the spray pattern was set to “criss‐cross” and nozzle height was appointed to 40 mm by default (technical details are presented in Table 1). Settings were chosen to ensure homogenous layers of matrix and small, evenly sized crystals. Matrix density (W, μg/mm^2^) is presented in Table 1 and was calculated by Supplementary Equation S1.

**TABLE 1:** Overview of tested matrix deposition protocols for peptide detection in prostate tissue by MALDI MSI

	CHCA matrix deposition parameters								
Method	T (°C)	NP	C (mg/mL)	FR (μL/min)	V (mm/min)	TS (mm)	Matrix density W (μg/mm^2^)	Solvent (% v/v)	Sprayer
M1	75	10	7	60	1200	2	1.8	50% ACN, 0.2%TFA	HTX
M2	75	4	7	75	1200	2	1.4	50% ACN, 0.2%TFA	HTX
M3	75	4	10	120	1300	2	2.0	50% ACN, 0.2%TFA	HTX
M4	80	6	7	100	1300	2	1.6	50% ACN, 0.2%TFA	HTX
M5^[^ ^4^, ^39^ ^]^	−	10	5	37	1390	2	0.7	50% ACN, 0.2% TFA	SunCollect

Abbreviations: CHCA = α‐Cyano‐4‐hydroxycinnamic acid, T = temperature, NP = number of passes, C = matrix concentration, FR = flow rate, V = velocity, TS = track spacing, W = NP×C×FR/V×TS, ACN = acetonitrile and TFA = trifluoroacetic acid.

### 2.9 MALDI MSI data acquisition

MALDI MSI was performed using three different rapifleX MALDI‐TOF Tissuetyper Bruker Daltonics instruments (one at NTNU, Trondheim, Norway; and two at University of Maastricht, Netherlands) according to equipment availability. The measurement regions were defined a few μm outside of the tissue section borders, in the matrix area and at the CytC spot. Data acquisition was performed in positive ion reflector mode at an *m/z* interval of 600–3000 using MALDI‐TOF MSI equipped with a 3D‐smartbeam laser (Nd:YAG with wavelength 355 nm) repetition rates ≤10 kHz. The laser was set to a single small raster size of 40 μm with a scan range of 30 μm x 30 μm. Spectra were accumulated from 800 laser shots fired at 10 kHz with a sampling rate of 1.25 GS/s. Red phosphorus was used as an external mass calibrant and laser power was adjusted to each slide. Data was processed during acquisition with TopHat baseline correction and Savitsky–Golay smoothing. After measurement with MALDI MSI, the very same sections were either stained by hematoxylin and eosin (H&E) or by using hematoxylin, eosin and saffron (HES) before the slides were digitally scanned and coregistered in flexImaging (v.5.0).

### 2.10 Data Processing

The CytC region was used as verification of trypsin digestion. If CytC tryptic peptide peaks, such as *m/z* 1168.6±0.6 Da (peptide sequence TGPNLHGLFGR), were present, further analysis was performed. Each average spectrum from the defined on‐tissue regions of interest (ROIs) was initially assessed by visual subjective inspection in flexImaging v.5.0 and peaks of interest were noted for later analyses. The total ion current (TIC) normalized average spectra were baseline corrected and matrix spectra were subtracted from on‐tissue spectra in mMass v.5.5.0 [31]. Peak picking was performed with a minimum S/N threshold of 3.0, a minimum relative intensity threshold of 0.1, a peak picking height of 100%, and isotopic peaks were removed. Acquisition data were imported and processed in SCiLS Lab (v.2019b SCiLS). Manual visual evaluation of every peak in the spectra was performed in SCiLS to ensure that deisotoping was achieved correctly, and that duplicate peaks and peaks with abnormal shapes were excluded, creating a postprocessed peak list for further analysis. A manual count of peaks in the postprocessed spectra were addressed as “number of peaks.” The forthcoming results in this paper is based on this postprocessed peak list. The “number of excluded peaks” was the sum of peaks subtracted from the preprocessed peak list as they either clearly belonged to the matrix, were isotopic peaks and/or technical noise.

### 2.11 Peptide identification with MS/MS

For peptide identification we used two methodological approaches. First, an Orbitrap Elite™ Hybrid Ion Trap‐Orbitrap Mass Spectrometer (Thermo Fisher Scientific GmbH, Bremen, Germany) coupled to a MALDI/ESI Injector (Spectroglyph LLC, Kennewick, USA) was used to acquire accurate mass. Separate prostate sections were covered with three layers of 2,5‐dihydroxybenzoic acid (DHB) matrix solution (50 mg/mL in 50% (v/v) ACN incl. 0.2% (v/v) TFA) at 30°C with a flowrate of 0.1 mL/mL (1200 mm/min velocity, track spacing: 1 mm, drying time: 10 seconds) using the HTX sprayer (W = 12.5 μg/mm^2^). The run was performed in data‐dependent acquisition (DDA) MS/MS mode in the ion trap [32]. However, since achieving high enough fragment ion signal for MS/MS identification is challenging with MALDI MS/MS due to lower precursor ion signal generated with MALDI [33, 34] only the high mass resolution mean mass spectrum was used for further data analysis.

LC‐MS/MS on peptide extracts was used for peptide sequencing. Three fresh frozen serial sections from the same prostate tissue sample were treated with the ice‐cold EtOH+H_2_O wash, heated at 95°C for 5 min and sprayed with trypsin (T2 protocol in Supplementary Table S1) and incubated overnight at 37.5°C. Peptides were extracted from the top of two of the tissue sections with 2% ACN 0.1% acetic acid and 5% ACN 0.1% acetic acid solutions as described by Drake et al. [35]. Tryptic peptides solutions from these two serial tissue sections were pooled, vacuum dried (SpeedVac, ThermoFisher) and dissolved in 15 μl 0.1% formic acid prior to LC‐MS/MS. LC‐MS/MS was performed on a timsTOF Pro (Bruker Daltonics) connected to a nanoElute (Bruker Daltonics) HPLC. Peptides were separated on a C18 column (Bruker FIFTEEN, Bruker daltonics) using a linear gradient from 0.1% formic acid to 37% ACN, 0.1% formic acid for 50 min at 300 nl/min. The timsTOF instrument was operated in the DDA PASEF mode with 10 PASEF scans per acquisition cycle and accumulation and ramp times of 100 ms each. The “target value” was set to 20 000 and dynamic exclusion was activated and set to 0.4 min. The quadrupole isolation width was set to 2 Th for *m/z* < 700 and 3 Th for *m/z* > 800. The third serial section was covered by CHCA matrix (protocol M1, Table 1) imaged with MALDI‐TOF MSI and peak picking was perfomed as described above. These peaks were matched with MALDI‐Orbitrap data to obtain high accuracy masses.

Peptide identification was performed for the HPLC‐timsTOF MS/MS data using MaxQuant (MQ, version 2). This approach used the *m/z*, ion intensites (area under peak), retention time and ion mobility dimensions to identify and sequence peptides through the DDA LC‐TIMS‐MS/MS Data workflow [36]. Allowed mass error was the default 10 ppm in MQ. The following search parameters were incorporated for both MALDI‐Orbitrap and HPLC‐timsTOF data in MQ: enzyme specified as Trypsin/P (max 2 missed‐cleavages allowed), acetylation of protein N‐terminal, oxidation of methionine, and deamidation of asparagine/glutamine as dynamic post‐translational modifications (max 5 per peptide). The Human proteome including isoforms downloaded June 2021 from Uniprot (Proteome ID UP000005640) along with MQ internal contaminant sequences were used as a database for search using the MQ built‐in Andromeda‐engine [37]. The false discovery rate was 1% for both peptide and protein identifications and only unique peptides were used for final protein group identification. Peptide identifications from MQ were compared to the list of peaks detected in MALDI‐TOF MSI (described in the paragraph above) and MALDI‐Orbitrap. Peptides with < 5 ppm mass error (MALDI‐Orbitrap data compared to theoretical *m/z*) were considered true matches. We note that the LC‐MS/MS and MALDI‐TOF MSI experiments were performed on serial sections of the same sample, whereas the MALDI‐Orbitrap data was acquired on a serial section from a different sample.

### 2.12 Quality evaluation

The evaluation measures used to determine the optimal method were: Number of detected tissue‐specific peaks (number of peaks), number of excluded peaks, percentage of high mass peaks (> *m/z* 2000), average signal‐to‐noise ratio (S/N), mass spatial localization score (LS) and signal intensity of selected peptides. The number of detected tissue‐specific peaks and excluded peaks were determined through the peak‐picking process described in the “Data processing” section. The percentage of high mass peaks was the percentage of tissue‐specific peaks found above *m/z* 2000. The S/N was calculated (average for all peaks) in mMass after baseline correction. The mass spatial localization was estimated according to a designated visual scoring system (ranging from 0–10) where the scores corresponded to how well the signal was detected on tissue; 0 = signal completely outside tissue, 5 = signal equally outside and on‐tissue, 10 = strong defined signal on‐tissue (Supplementary Figure S2). A total LS was determined for each tissue section, where the average score was measured from five prominent masses (*m/z* 868, 1095, 1198, 1562 and 1706). To evaluate the signal intensity of selected peptides between the different measurements, the log_10_ transformed average intensities (height of the peak) of four peptides (*m/z* 868, 976, 1542 and 1562) were calculated for the mean spectrum for each section. These peptides were selected as they showed varying intensity across all the different sections and histology and thus limited bias toward sample origin or stroma/epithelium composition. A quality evaluation (QE) scoring system was constructed based on these measures, where each protocol received a score between 0 (poor result) and 10 (excellent result). For each measure, the values were first transformed to a scale of 0–10 (for details see Supplementary Equation S2). The total QE‐score was then calculated as the weighted average of the individual QE scores (see Supplementary Equation S3). The weighting factors for each measure were determined by ranking the measures according to their importance for MALDI MSI. We defined 3 ranks: High importance (percentage of high mass peaks (> *m/z* 2000), mass spatial localization), medium importance (number of excluded peaks, signal intensity of selected peptides), and low importance (average S/N, number of peaks). The weights were set to be equal for all members of one rank and two times the value of the weight of the previous rank.

### 2.13 Statistics

Established quality parameters were checked for normal distribution and means were compared by using two‐tailed independent Student's t‐test among the different testing steps using the total QE‐score with a significance level of p≤0.05 in SPSS (IBM ® SPSS® Statistics, version 27) and Microsoft® Excel® for Microsoft 365 MSO (version 2008). For non‐normally distributed samples, nonparametric Mann–Whitney tests were performed in SPSS.

## 3 RESULTS AND DISCUSSION

**FIGURE 2:**
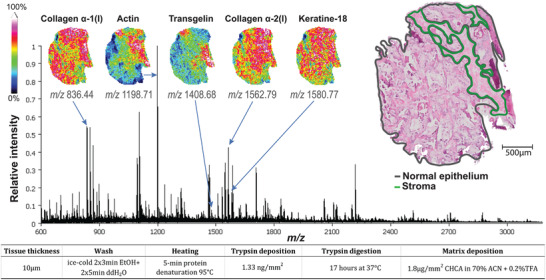
Representative peptide spectrum after the optimal sample preparation protocol on fresh frozen prostate tissue measured by MALDI TOF MSI. The spatial distribution of five features that were identified as peptides by MS/MS are presented: collagen α‐1(I) (*m/z* 836.44), actin (*m/z* 1198.71), transgelin (*m/z* 1408.68), collagen α‐2(I) (*m/z* 1562.79) and keratin‐18 (*m/z* 1580.77)

We have developed an optimized protocol for spatial detection of proteolytic peptides in fresh frozen prostate tissue using MALDI‐TOF MSI where a representative spectrum with selected peptide masses is shown in Figure 2. Peptide detection on fresh frozen tissue has generally been considered challenging within the imaging community [38] in comparison to MSI measurement of metabolites or lipids as those protocols require fewer sample preparation steps. A selection of previously published MALDI MSI protocols analyzing proteomics on fresh frozen tissue was used as a starting point and iteratively adapted by trial and error and further compared to detect spatial peptide distribution [2, 9, 12, 13, 15, 21, 39]. The key optimization steps tested were washing, heating, tryptic digestion, and matrix application (Supplementary Table S2). The QE‐scores of the 25 unique methods are shown in Supplementary Figure S3 and Supplementary Table S3. The optimal peptide protocol (method 6) included ice‐cold EtOH+H_2_O wash, heating for 5 min at 95°C, overnight (17 h at 37°C) digestion using trypsin density of 1.3 ng/mm^2^ and 7 mg/mL CHCA matrix applied with 10 layers using 0.06 mL/min flowrate. This optimal protocol resulted in an average number of peaks of 167 ± 56 (30 ± 38 peaks excluded) where 30% of the peaks were found above *m/z* 2000 which is considered challenging for peptide detection. This method harbored an average S/N of 7.3 ± 1.2, where the spatial peptide distribution corresponded well to the annotated epithelial and stromal areas in the histology sections (Figure 2) as the localization score was high (LS = 7.73 ± 0.81, 41% above the average of all methods). Including a heating step after washing was found to be critical for obtaining the best quality MSI spectra from fresh frozen prostate tissue. The heating contributed to detection of more masses (especially masses above *m/z* 2000) and an increased higher spatial localization (Figure 2) in comparison to other protocols tested.

### 3.1 Detailed protocol optimization step‐by‐step

#### 3.1.1 Tissue preparation (step 1)

Cryosectioning of fresh frozen prostate tissue from tissue cores of 10 μm thickness provided a more clean‐cut tissue surface than 4 μm sections, as the tissue sections of 4 μm had a more crocked or irregular shape which subsequently affected the quality of the MSI images (Supplementary Figure S4). This could be explained by the texture and structure of the prostate tissue, the freezing or by the quality of the sectioning, as thicker sections are technically easier to cut. However, if possible without the expense of section quality, thinner sections can improve analyte intensities in MALDI MSI experiments [40]. Thickness of 4 μm is more frequently used with FFPE tissue [41], while 10 μm thickness is more common for fresh frozen tissue in MSI experiments [42]. The majority of the experiments (21 of 25 protocols) were performed with a thickness of 10 μm due to higher sectioning quality observed during testing.

#### 3.1.2 Optimizing tissue wash for improved spatial peptide localization (step 2)

**FIGURE 3:**
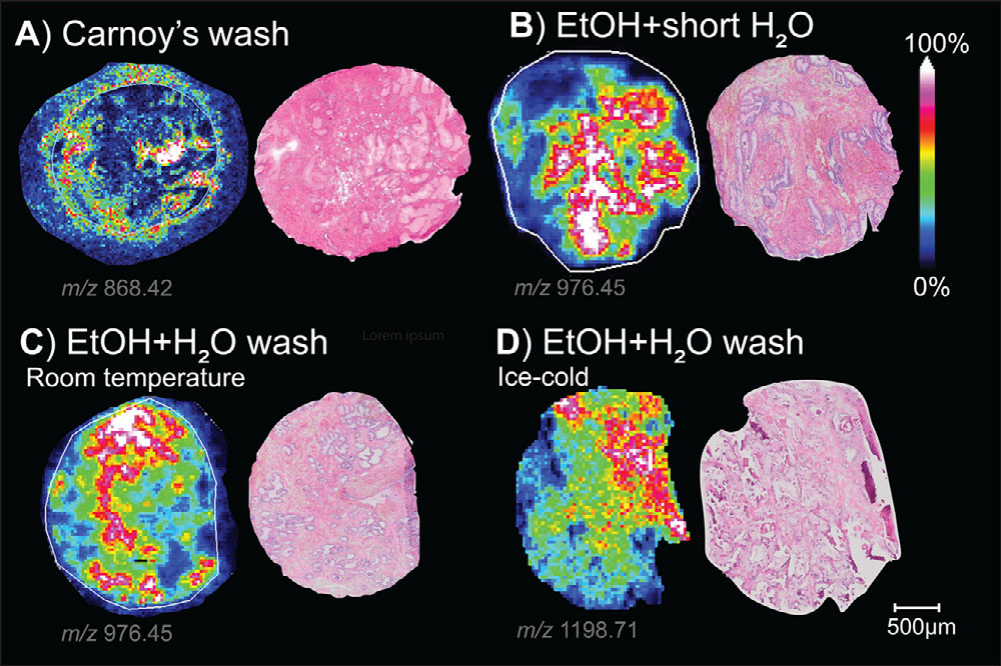
MALDI MSI images and corresponding HES of fresh frozen prostate tissue sections using various washing protocols where the masses with the highest S/N are shown from different tissue sections (due to different histology, the *m/z* values with the highest S/N vary between each image). A) Carnoy's wash, B) RT EtOH+H_2_O wash, C) RT EtOH+ H_2_O wash or D) ice‐cold EtOH+ H_2_O wash

A selection of four washing procedures were tested and resulting MALDI images are presented in Figure 3. We also initially tested other published washing procedures [5, 6, 39], but these were excluded from our study due to detrimental effects on the final HE images.

Overall, methods using EtOH+H_2_O wash gave on average 39% higher QE scores than methods using Carnoy's wash, which additionally resulted in alterations of the tissue morphology (Figure 3A). Therefore, we further focused on the optimization potential of EtOH+H_2_O wash, initially by reducing the time of the H_2_O steps and later by alternatively using ice‐cold EtOH and H_2_O with the purpose of minimizing delocalization. We observed improved spatial localization using the ice‐cold EtOH+H_2_O wash in matched sample comparison (Supplementary Figure S5). However, on average, all three EtOH+H_2_O washes led to comparable localization and total QE scores when only including samples undergoing heat treatment in Step 3.

#### 3.1.3 Brief heat treatment improved signal intensity (step 3)

**FIGURE 4:**
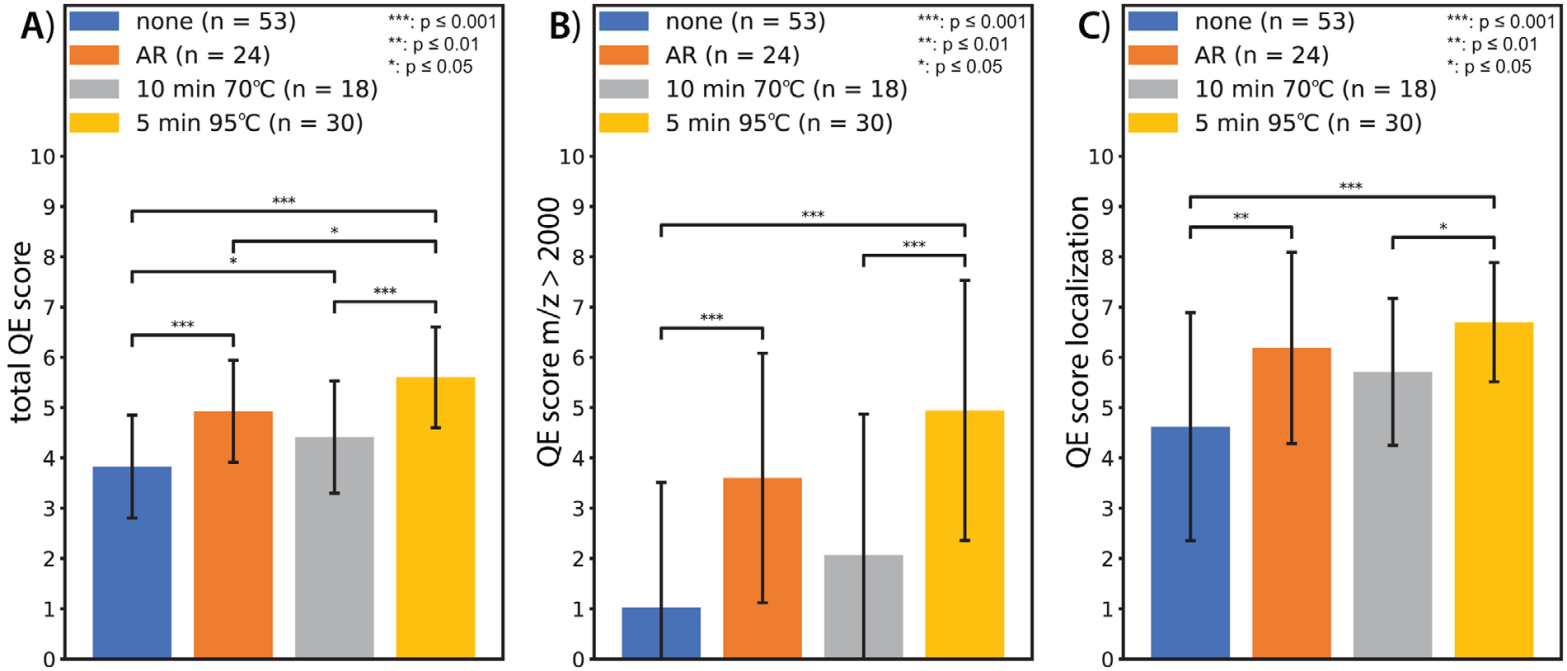
Evaluation of adding a heating step to the sample preparation protocol. A) Total QE‐score for the respective methods, B) percentage of masses detected above *m/z* 2000, and C) average localization score. Without any heating step (blue, n = 53), with antigen retrieval (AR, orange, n = 24), protein denaturation step 10 min at 70°C (gray, n = 18), and protein denaturation step 5 min at 95°C (yellow, n = 30). Error bars represent standard deviation. Significance levels are indicated as * = p ≤ 0.05; ** = p ≤ 0.01; *** = p ≤ 0.001

Of the four different heating procedures tested, the 5 min at 95°C protein denaturation step where the slides were quickly brought to 95°C under water‐vapor had a significantly higher QE‐score in comparison to protocols with no heat treatment (p ≤ 0.001; Figure 4A), 10 min at 70°C in a humid chamber (p ≤ 0.001; Figure 4A) and in comparison to antigen retrieval (AR; p ≤ 0.05; Figure 4A). Of note, the sections run with AR showed a higher variability in the localization score between sections than 5 min at 95°C or 10 min at 70°C (Figure 4C) and typically lacked mass in the higher mass range (> *m/z* 2000; Figure 4B), resulting in lower reproducibility. The protein denaturation step of 10 min at 70°C scored higher (p ≤ 0.05) than no‐heating, and AR resulted in significantly (p ≤ 0.001) higher scores than no heating and slightly higher scores than 10 min at 70°C protocol (Figure 4A). Completely leaving out the heating step from the sample preparation procedure, gave the lowest localization scores (Figure 4C) and typically lacked heavier masses (> *m/z* 2000 ; Figure 4B). Our results clearly show that including a heating step was beneficial for detection of peptide signals in general and by using a higher temperature of 95°C for 5 min, increased the QE scores. Although the AR protocol provided data of higher quality than not using heating at all or heating for 10 minutes at 70°C, the observed higher variability in localization and lack of higher masses, makes this method less reliable. This could be explained by the harsh conditions of submerging the tissue sections in a boiling acidic solution at 121°C and the much longer incubation time of approximately 30 minutes. Rapid heating (95°C for 5 min), also called heat‐stabilization, is a common treatment of fresh tissue to inactivate proteins [43]. Heating the tissue results in irreversible denaturation of proteins through unfolding which prevents autolytic degradation and facilitates the access of trypsin for a more efficient digestion. The same effect was also observed by Zheng and DeMarco, where heating plasma samples at 99°C for 5 minutes prior to trypsin digestion resulted in higher peptide signals from certain proteins while ensuring higher peptide stability compared to using no heating [44]. In our study, ice‐cold tissue wash combined with a subsequent heating step at high humidity (method 6, Supplementary Table S3) gave the highest QE scores for localization and peptide signal. Although comparing the different washing steps could not demonstrate a clear benefit for ice‐cold EtOH wash, we suggest that proteins might be fixed at their inherent position during the ice‐cold wash and then being unfolded in‐place through the rapid heating step, making them more accessible for enzymatic cleavage and thereby producing a higher number of peptides at their specific location.

As the protein denaturation step (heating step) was included later in the timeline, not all washing procedures were tested with this step. Therefore, we cannot exclude the possibility that the other washing procedures could be more effective when also including a heating step for peptide detection on fresh frozen prostate tissue sections.

#### 3.1.4 Low trypsin density and overnight incubation produced high‐quality measurements (step 4–5)

There was no remarkable difference between applying slightly higher or lower trypsin density (1.3 ng/mm^2^ vs. 6.7 ng/mm^2^) judging from the QE scores of samples undergoing the heat treatment of 95°C for 5 min (Supplementary Figure S6). As using less trypsin is more cost‐efficient, we preferred the trypsin application method T2 resulting in trypsin density of 1.3 ng/mm^2^. While others have suggested increased S/N with increasing trypsin concentration for FFPE kidney, heart and aorta tissue samples [14], this was not the case in our study. A higher trypsin concentration may also increase the degree of autolytic peptides from the trypsin, which could give rise to more unwanted signal interference [45].

**FIGURE 5:**
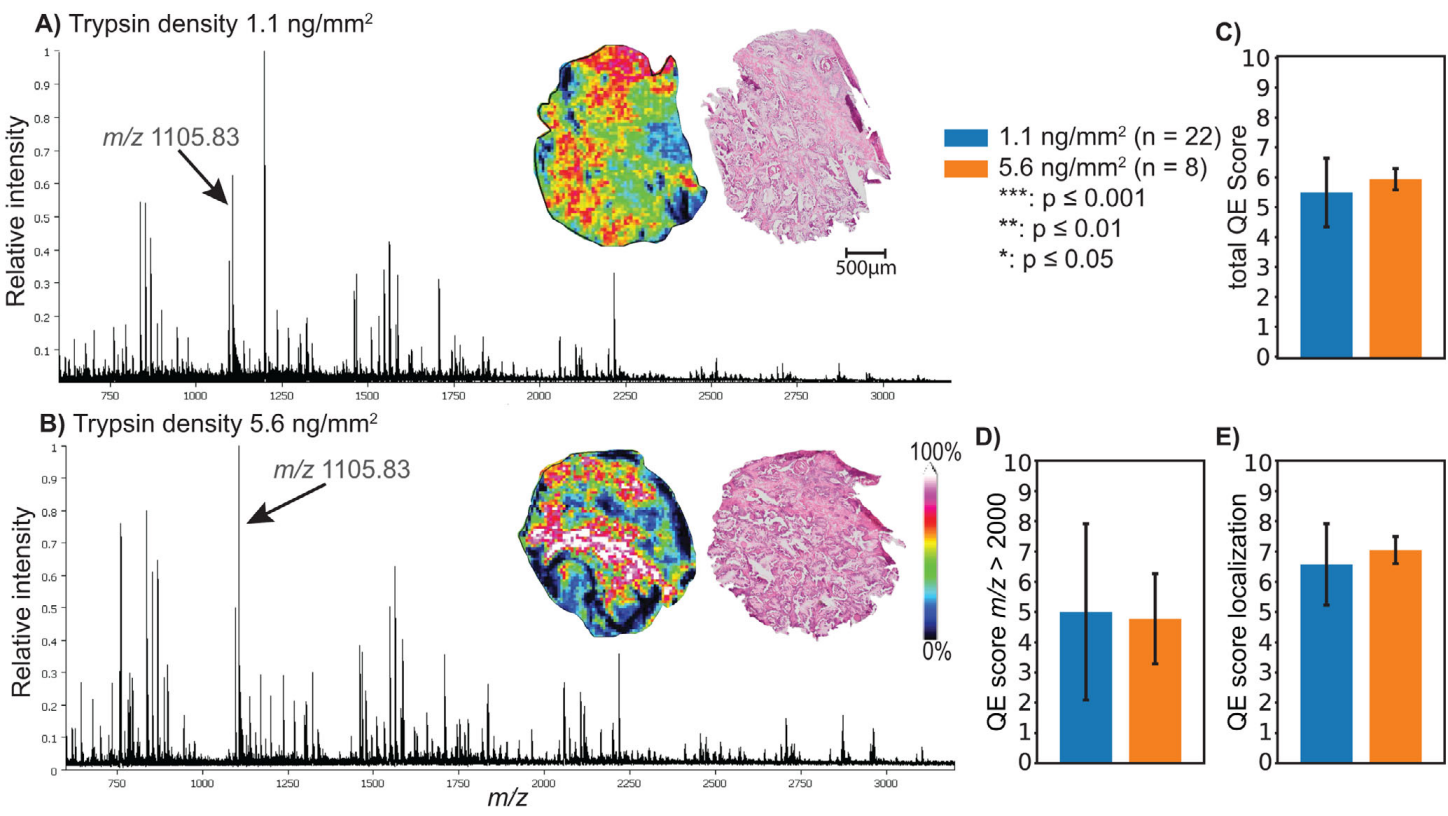
Optimization of trypsin digestion showing mean mass spectra of tryptic digestion routines at A) 2 h at 50°C and B) 17 h at 37°C showing C) total QE score, D) QE score percentage masses above *m/z* 2000 and E) localization QE score. Error bars represent standard deviation. Significance levels are indicated with p‐values with a threshold of α = 95%; * = p≤0.05; ** = p ≤ 0.01; *** = p ≤ 0.001

When comparing all samples with short digestion (2 h at 50°C) to all samples with overnight digestion (17 h at 37°C), the total QE score for overnight digestion was on average significantly higher (22%, p *≤* 0.001, Figure 5C). Further, short digestion led to a higher number of noisy background peaks with similar intensities and a typical distribution of 1 Da spacing (Figure 5A). Short digestion offered highly localized signals, though failed to detect peptides on tissue, especially above *m/z* 2000, which resulted in spectra of lower quality (Figure 5A). MSI spectra of sections with overnight digestion showed a significantly higher percentage of peaks belonging to the upper mass range of the MSI spectrum (on average 215% higher score, p *≤* 0.001; Figure 5D).

#### 3.1.5 Matrix solution and application (step 6)

Developing the optimal matrix application protocol is crucial to ensure analyte extraction at their spatial localization. Due to the many parameters included in the complete sample preparation procedure, we decided to only consider samples that underwent either AR or 5 min 95°C. Thus, we compared four (M1, M3, M4, M5) of the five different spray routines tested (Table 1) with each other. Methods M1, M3, M4 with a matrix density of 1.8, 2.0, 1.6 μg/mm^2^, respectively, resulted in on average 26–33% (p ≤ 0.05) higher total QE scores than method M5 with a matrix density of 0.7 μg/mm^2^ (example images and spectra in Supplementary Figure S7). Of note, the higher matrix densities of methods M1, M3, and M4 resulted in significantly higher QE scores for *m/z *above 2000 and intensities of selected peptides than method M5 (on average 140% and 220% higher, p ≤ 0.05 and p ≤ 0.001), respectively. The localization score was on average the same for all methods. Apart from the matrix density, the most obvious difference between the methods was that M1, M3, and M4 utilized an HTX sprayer and M5 the SunCollect sprayer. It remains inconclusive if the device itself also plays a role in the observed worse detectability of larger molecules or if this effect can only be attributed to the lower matrix density.

### 3.2 Peptide identifications

Of the masses identified from LC‐MS/MS, 2 723 tryptic peptides from 838 proteins, a total of 24 pepetides from 12 proteins were also detected with MALDI MSI (Table 2). The majority of the identified peptides originated from collagens. Collagen is an important building block of the extracellular matrix and is a prominent part of stromal tissue. Recent findings have addressed different types of collagens to be active components of reactive stroma in prostate cancer which contributes to disease progression [46, 47]. Actin was another protein identified through detection of several peptides. As actin is a key component of muscle fibers and prostate stroma contains a high proportion of smooth muscle cells, this was a reasonable finding. Actin is also suggested as a prognostic marker in prostate cancer [48] and is along with collagens an interesting protein for further investigation as a clinical marker for prostate cancer. For the remaining 9 proteins identified, we could detect and assign one peptide each. Nevertheless, presence in prostate tissue is reasonable for all of them and functional roles in cancer have been reported previously [49, 50, 51, 52, 53, 54]. The MS/MS results demonstrate that peptides of biological and clinical interest could be detected with our optimized sample preparation protocol.

**TABLE 2:** A total of 24 peptides originating from 12 proteins could be detected with MALDI‐TOF MSI, MALDI‐Orbitrap and were identified through LC‐MS/MS

Detected*m/z* [M+H]+	Theoretical *m/z* [M+H]+	Mass err (ppm)	Peptide sequence	Protein (Accession ID)
945.552 976.449 1198.705	945.552 976.448 1198.706	−0.21 −0.82 0.17	AVFPSIVGR^1^ AGFAGDDAPR AVFPSIVGRPR	Actin, cytoplasmic (P63261) and/or Actin, smooth muscle (P63267)
1087.564	1087.563	−1.38	IDQNVEELK	Apolipoprotein A‐IV (Q15969)
836.438 868.428 886.438 945.439 1297.613	836.437 868.427 886.438 945.438 1297.613	−0.60 −0.58 −0.68 −0.21 0.15	GPAGPQGPR GEAGPQGPR^2^ GSEGPQGVR QGPSGASGER^1^ GESGPSGPAGPTGAR	Collagen α‐1(I) (P02452)
771.411 785.391 809.438 840.469 868.464 1235.613 1562.795	771.410 785.390 809.438 840.469 868.464 1235.613 1562.792	−0.39 −1.40 −0.37 −0.24 −0.58 −0.24 −1.67	GASGPAGVR GDQGPVGR GHAGLAGAR GVVGPQGAR GPSGPQGIR^2^ GEAGAAGPAGPAGPR^3^ GETGPSGPVGPAGAVGPR	Collagen α‐2(I) (P08123)
1303.603	1303.599	−2.99	NALESYAFNM(Ox)K	Heat shock 70 kDa protein (P0DMV8 and/or P0DMV9)
1207.570	1207.570	−0.17	ASYAQQPAESR	Basement membrane‐specific heparan sulfate proteoglycan core protein / perlecan (P98160)
1580.767	1580.766	−0.51	PVSSAASVYAGAGGSGSR	Keratin‐18 (P05783)
1302.689	1302.690	0.69	SVSLTGAPESVQK	Far upstream element‐binding protein 2 (Q92945)
1742.742	1742.746	2.47	LESGGSNPTTSDSYGDR	Protein phosphatase 1 regulatory subunit 12B (O60237)
1337.682	1337.681	−0.97	AVVVHAGEDDLGR	Extracellular superoxide dismutase (Q08420)
1408.676	1408.675	−0.50	GASQAGMTGYGRPR	Transgelin (Q01995)
1125.529	1125.531	2.04	VEYSEEELK	X‐ray repair cross‐complementing protein 6, Ku70 (P12956)

A maximum mass error of 5 ppm between detected *m/z* value from high‐resolution MALDI‐Orbitrap and theoretical *m/z* was used. Oxidation of methionine is indicated as “M(Ox)” in the peptide sequence.

^1^ Peptides of actin (*m/z* 945.552) and collagen α‐1(I) (*m/z* 945.439) likely overlaps and is detected as one peak in the MALDI‐TOF MSI data.

^2^ Peptides of collagen α‐1(I) (*m/z* 868.428) and collagen α‐2(I) (*m/z* 868.464) likely overlaps and is detected as one peak in the MALDI‐TOF MSI data.

^3^ This mass may also be peptide sequence AAQDRDQIYR arising from transformer‐2 protein homolog Β (P62995)

### 3.3 Applicability of method

Although the described optimizations of sample preparation for peptide detection are performed on fresh frozen prostate tissue, the process of developing this protocol could be relevant for peptide detection in other tissue types or organs. From our experience, some degree of protocol optimization is always necessary and this study provides a guide on the critical steps to optimize. We show that the heating step at high humidity (as opposed to submerged in solution) was a necessity for improved MSI peptide detection and localization. Further, tissue wash was the second most crucial step to optimize for MALDI MSI peptide detection, followed by trypsin application and matrix deposition. The optimal washing procedure is also what we anticipate that will vary the most across different tissue types due to different molecular compositions and functional structure as has been pointed out before in intact protein analysis [21, 39, 41]. For instance, for tissues such as breast and liver, containing a high concentration of fat, stronger organic solvents may be required in order to wash away fatty compounds to improve peptide detection.

## 4 CONCLUDING REMARKS

This study presents an optimal sample preparation protocol for spatial peptide detection on fresh frozen prostate tissue using MALDI MSI. Most importantly, we have shown that a 5 min heating step at 95°C, directly performed after tissue wash, enhances the signal intensity on tissue and increases the quality of the MSI spectra. This protocol successfully managed to increase the number of detected peptides on tissue and provide their inherent spatial distribution minimizing delocalization. We have demonstrated that proper sample preparation is crucial to achieve high‐quality MALDI MSI measurements ensuring reproducibility, which will facilitate future experiments aiming for the discovery of novel biomarkers for aggressive prostate cancer. Additionally, we provide an optimized protocol that highlights the critical performing steps in tryptic peptide analysis that could be useful for other protocol set‐ups within the MALDI imaging community and also for general LC‐MS peptide analysis.

## CONFLICT OF INTEREST

The authors declare no conflict of interest.

## Supporting information



Supporting Information

## Data Availability

Due to data size and privacy regulations, the raw data that support the findings of this study are not publicly deposited, but are available on request.
