# Supplementary material for: An optimized MALDI MSI protocol for spatial detection of tryptic peptides in fresh frozen prostate tissue
Source: Proteomics. 2022 Mar 3;22(10):2100223. doi: 10.1002/pmic.202100223 (PMC9285595; doi:10.1002/pmic.202100223)
Supplement: Supplementary file 1 — Supporting Information [file PMIC-22-0-s001.pdf]

An optimized MALDI MSI protocol for spatial detection of tryptic peptides in fresh frozen prostate tissue

Therese S. Høiem[1*], Maria K. Andersen[1], Marta Martin-Lorenzo[2], Rémi Longuespée[3], Britt S.R. Claes[2], Anna Nordborg[4], Frédéric Dewez[2], Benjamin Balluff[2], Marco Giampà[5], Animesh Sharma[5,6], Lars Hagen[5,6,7], Ron M.A. Heeren[2], Tone F. Bathen[1,8], Guro F. Giskeødegård[9], Sebastian Krossa[1], May-Britt Tessem[1,10*]

1. Department of Circulation and Medical Imaging, NTNU - Norwegian University of Science and Technology, Trondheim
2. Maastricht MultiModal Molecular Imaging Institute (M4I), Maastricht University, Maastricht
3. Department of Clinical Pharmacology and Pharmacoepidemiology, Heidelberg University Hospital, Heidelberg
4. Department of Biotechnology and Nanomedicine, SINTEF Industry, Trondheim
5. Department of Clinical and Molecular Medicine, NTNU - Norwegian University of Science and Technology, Trondheim
6. PROMEC Core Facility for Proteomics and Modomics, NTNU - Norwegian University of Science and Technology and the Central Norway Regional Health Authority Norway, Trondheim, Norway
7. Clinic of Laboratory Medicine, St. Olavs Hospital, Trondheim University Hospital, Trondheim, Norway
8. Department of radiology and nuclear medicine, St. Olavs Hospital, Trondheim University Hospital, Trondheim
9. Department of Public Health and Nursing, NTNU - Norwegian University of Science and Technology, Trondheim
10. Department of Surgery, St. Olavs Hospital, Trondheim University Hospital, Trondheim

Corresponding authors*: therese.s.hoiem@ntnu.no, may-britt.tessem@ntnu.no

Table of contents

Supplementary Figures

1. Supplementary Figure S1: ITO glass layout
2. Supplementary Figure S2: Localization scoring system
3. Supplementary Figure S3: QE-score overview
4. Supplementary Figure S4: Tissue thickness 4µm vs 10µm
5. Supplementary Figure S5. RT vs ice-cold EtOH+H_2O wash
6. Supplementary Figure S6. 0.02 vs 0.1 µg/µl trypsin concentration
7. Supplementary Figure S7. Optimization of matrix spraying

Supplementary Equations

1. Supplementary Equation S1: Spray density calculation
2. Supplementary Equation S2: QE-score transformation
3. Supplementary Equation S3: Total QE-score calculation

Supplementary Tables

1. Supplementary Table S1: Trypsin solution and deposition technical details
2. Supplementary Table S2: Overview of optimization steps for sample preparation
3. Supplementary Table S3: Overview of all the 25 sample preparation protocols and their average QE-scores

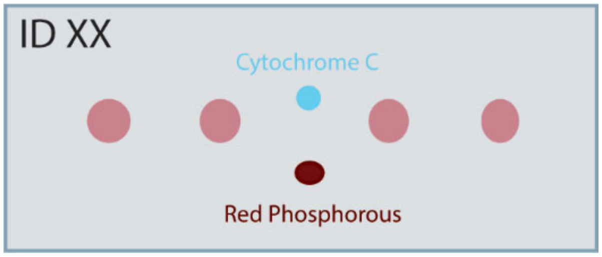

Supplementary Figure S1. ITO glass slide layout. Four fresh frozen sections of prostate tissue samples (⌀=3mm), visualized as red circles.

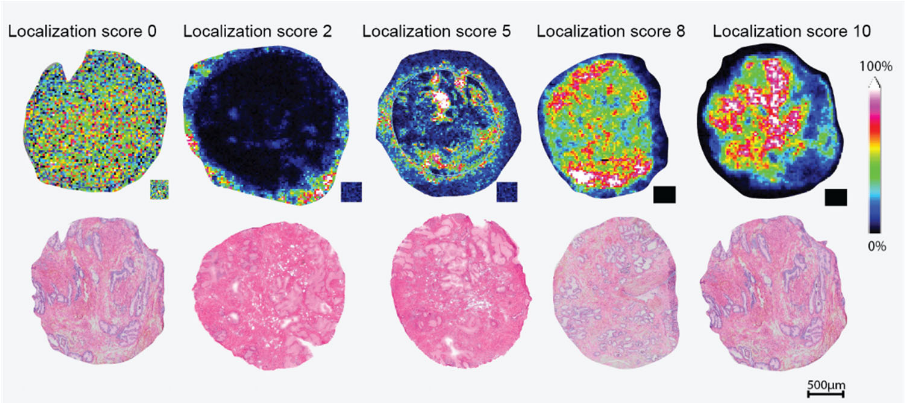

Localization score	Definition
0	All signal detected outside tissue, representing only technical noise or no signal detected
1-4	Signal detected partially outside tissue
5	Signal detected with equal parts outside and on tissue
6-9	Signal mainly detected on tissue, with minor outside tissue
10	Signal detected perfectly within borders of tissue

MALDI-TOF MSI	*m/z* 868.44	*m/z* 1095.5	*m/z* 1198.71	*m/z* 1562.79	*m/z* 1706.78	Total localization score

Supplementary Figure S2. Localization scoring system for spatial detection of peptides. Examples of MSI images of delocalization scores 0-10 are presented at the top based on the definitions described below. Five m/z values from each sample were chosen to represent the MSI spectra to evaluate the quality of MSI spectra and localization, and the average of these five were addressed as total localization score.

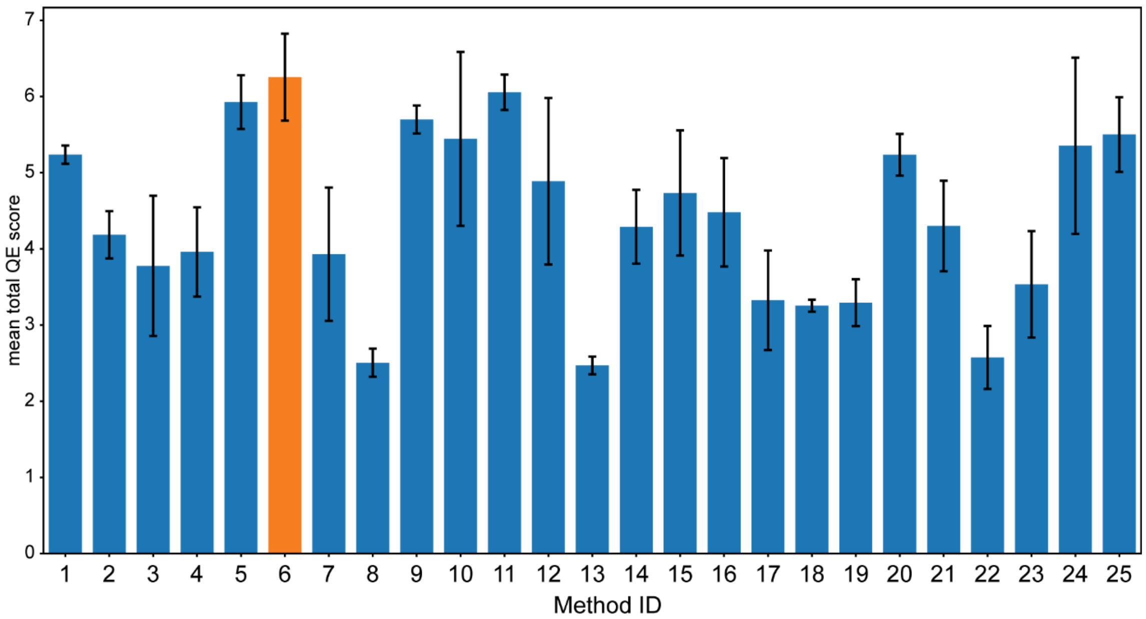

Supplementary Figure S3. Mean total quality evaluation score (QE-score) from 25 different sample preparation optimization protocols measured on fresh frozen prostate tissue using MALDI MSI. QE-scores were based on a weighted sum of the evaluation criteria number of peaks, number of excluded peaks, percentage masses detected above m/z 2000, S/N, localization score and peptide intensity scores, as described in the method section. The highest scoring protocol (method ID 6, details in Supplemental Table 2) according to the QE-score is marked in orange, and error bars represent standard deviation.

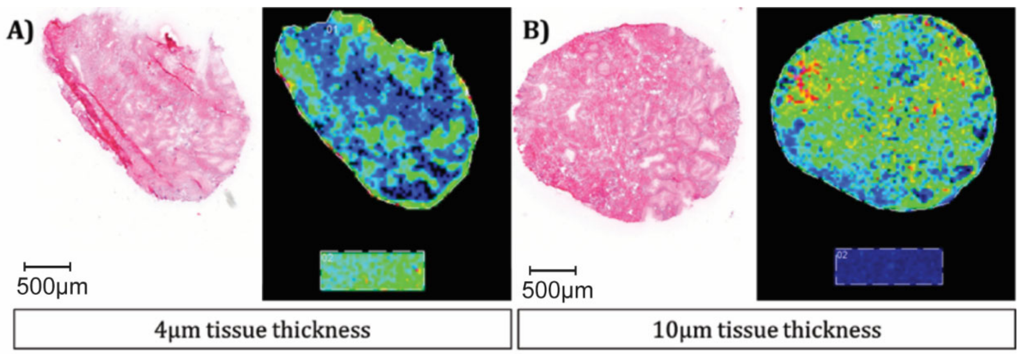

Supplementary Figure S4. HES stained prostate tissues (left) sectioned at either A) 4 μm or B) 10μm thickness with their corresponding MALDI MSI images of m/z 1325.789 Da ± 376.

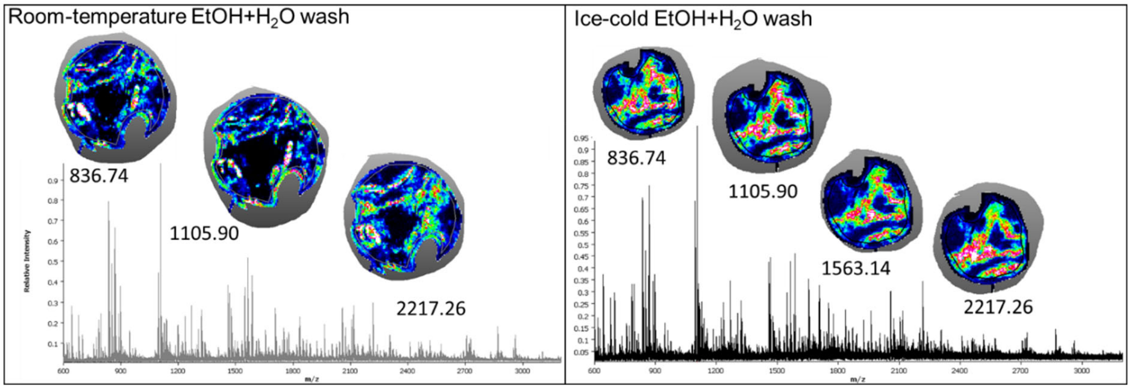

Supplementary figure S5. MSI images and spectra of representative samples of either RT or ice-cold EtOH+H_2O wash.

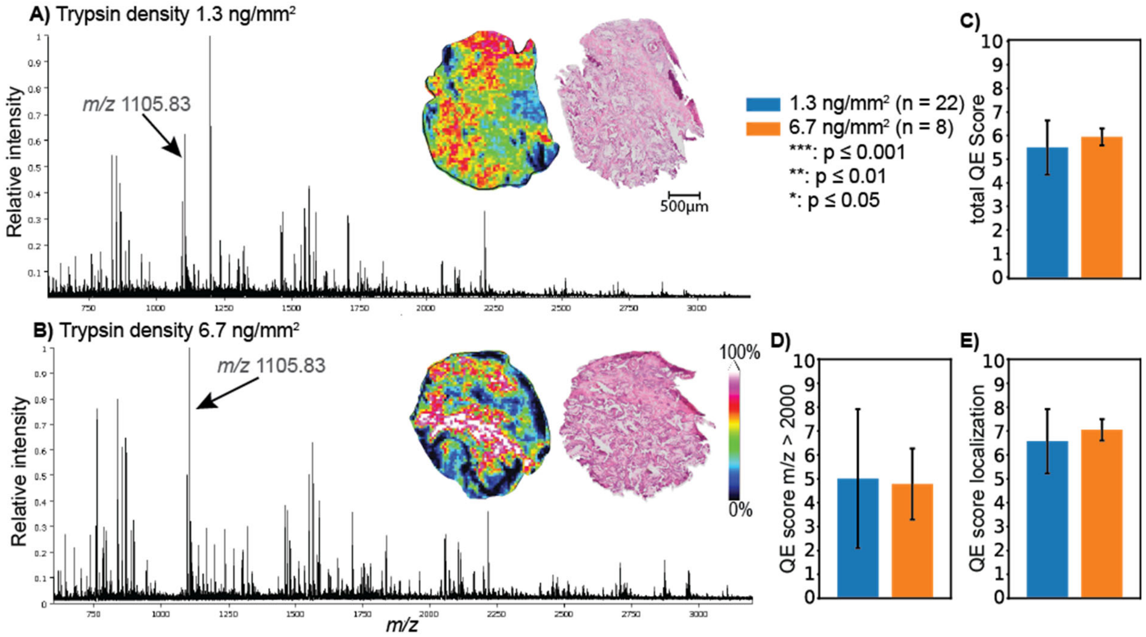

*Supplementary Figure S6. Optimization of on tissue trypsin density, showing mean mass spectra and corresponding HES stained section of A) low trypsin density (1.3 ng/mm^2) and B) high trypsin density (6.7 ng/mm^2). C) The total QE score, D) QE score for masses m/z > 2000, and E) the localization score for all samples undergoing heat treatment for 5 min at 95°C was on average the same for low and high density trypsin. Error bars represent standard deviation. Significance levels are indicated with p-values with a threshold of α=95%; *=$p \leq 0.05$; **=$p \leq 0.01$; ***=$p \leq 0.001$.*

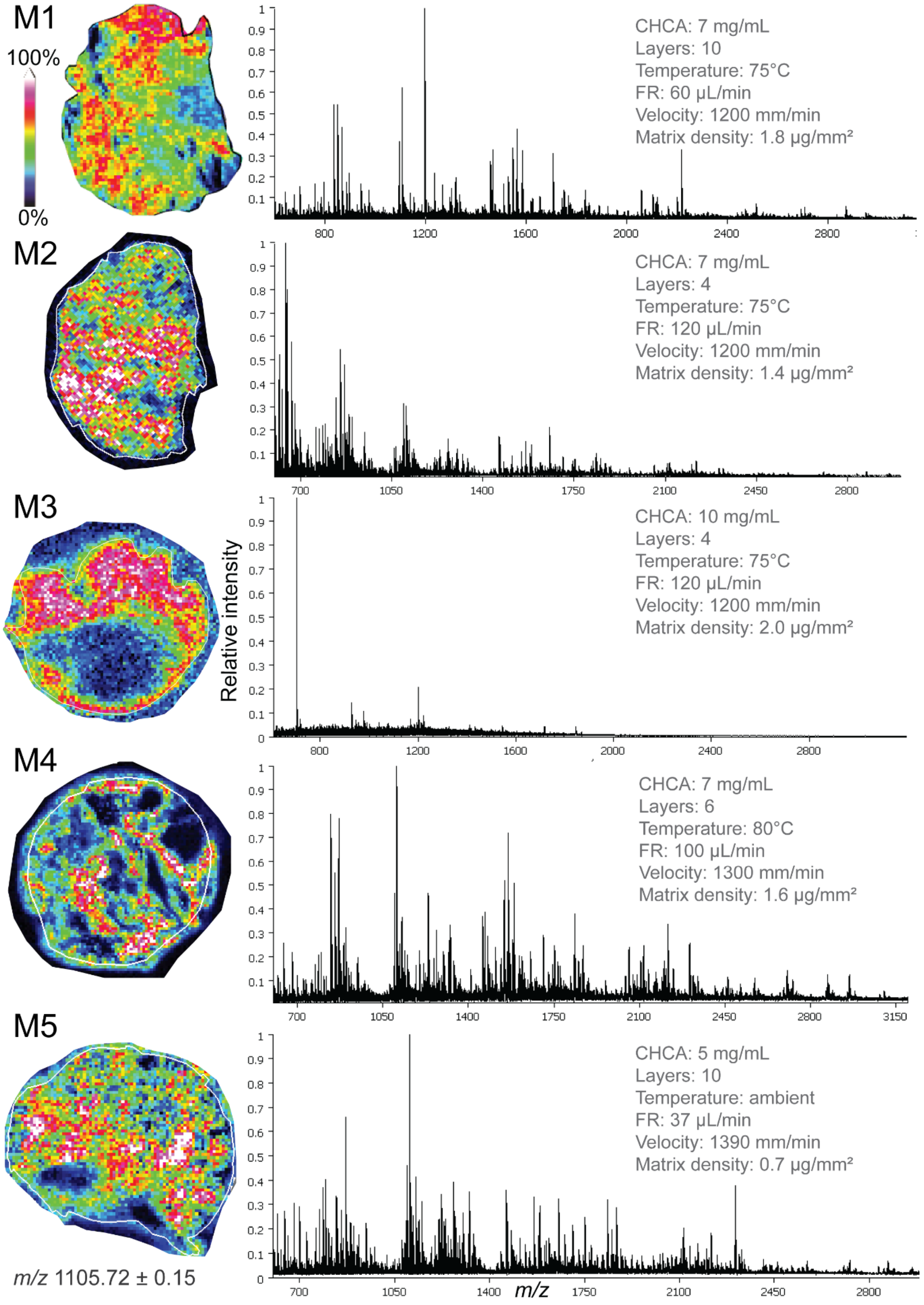

Supplementary Figure S7. Exemplary MS images and average spectra obtained after applying the five different matrix spray protocols (M1 – M5) for deposition of CHCA where M1, M3, and M4 had significantly higher QE-score than M5. All spray routines had a pressure of 10 psi, a spray nozzle height of 40 mm and a track spacing of 2 mm. The representative MSI images are based on m/z 1105.72 ± 150ppm. FR = flow rate.

Supplementary Equation S1. Density (W) equation for calculating trypsin and matrix application density.

$$W = \frac{\left(\text{number of passes} \times \text{solvent concentration} \left(\frac{\text{mg}}{\text{mL}}\right) \times \text{flow rate} \left(\frac{\mu\text{L}}{\text{min}}\right)\right)}{\text{nozzle velocity} \left(\frac{\text{mm}}{\text{min}}\right) \times \text{track spacing (mm)}}$$

Supplementary Equation S2. Each evaluation measure was expressed as a quality evaluation (QE) score according to the degree of success within the interval of 0-10, where 10 were determined as most successful, and 0 as worst. For each measure the values were transformed into a scale of 0-10 using the given equation.

$$QE_{i,n} = \begin{cases} 10 * \dfrac{(I_n - \min(I))}{\max(I) - \min(I)}, & i \neq excluded\ peaks \\ 10 - 10 * \dfrac{(I_n - \min(I))}{\max(I) - \min(I)}, & i = excluded\ peaks \end{cases}, with$$

$$I_n\ value\ for\ measure\ i\ for\ experiment\ n,$$

$$\max(I)\ maximum\ of\ all\ observed\ values\ for\ measure\ i,$$

$$\min(I)\ minimum\ of\ all\ observed\ values\ for\ measure\ i$$

Supplementary Equation S3. The total QE score was calculated as the weighted average of the QEs for each evaluation measure for each experiment. After normalizing the sum of all weights to 1, this resulted in the following weights used to calculate the total QE score: percentage of high mass peaks (>m/z 2000) w = 0.29, mass spatial localization w = 0.29, number of excluded peaks w = 0.14, signal intensity of selected peptides w = 0.14, average S/N w = 0.07, number of peaks w = 0.07.

$$QE_{total,n} = \frac{\sum_i w_i * QE_{i,n}}{\sum_i w_i}, with$$

$$w_i\ weight\ factor\ for\ each\ measure\ i,$$

$$QE_{i,n}\ score\ for\ each\ measure\ i\ for\ experiment\ n$$

Supplementary Table S1. Overview of spraying details for four different trypsin application methods. The optimal method is marked in bold.

Method	Trypsin concentration	Solvent	Trypsin application	Trypsin density	Sprayer
T1	0.02 µg/µL	Ice-cold H_2O	15 layers, 10 µL/min flow rate, Z:25 mm, D:1.0 mm, speed:900 mm/min	3.3 ng/mm^2	SunCollect
**T2**	**0.02 µg/µL**	**Ice-cold H_2O**	**8 layers, 30 µL/min flow rate, 1200 mm/min, 45°C, track spacing:3**	1.3 ng/mm^2	**HTX M5**
T3	0.1 µg/µL	Ice-cold H_2O	8 layers, 30 µL/min flow rate, 1200 mm/min, 45°C, track spacing:3	6.7 ng/mm^2	HTX M5
T4	0.1 µg/µL	Ice-cold H_2O	15 layers, 10 µL/min flow rate, Z:25 mm, D:1.0 mm, speed:900 mm/min	16.7 ng/mm^2	SunCollect

Supplementary Table S2. Overview of the optimization steps for the sample preparation protocol for peptide measurement by MALDI TOF MSI in fresh frozen prostate tissue. Optimal parameters are highlighted with "x" in the right-side column. Double "x" within each step means procedures are considered comparable. FR = flow rate.

Protocol parameters for optimization	Description	Optimal
**Step 1, Cryosectioning**		
Tissue thickness	4µm	
	10µm	x
**Step 2, Tissue washing**		
Carnoy's wash	Room temperature	
EtOH+H_2O wash	Room temperature	
Ice-cold EtOH+H_2O wash	EtOH = -80°C, H_2O = 4°C	x
EtOH+short H_2O wash	Room temperature	
**Step 3, Heating step**		
None		
Protein denaturation	10 min 70°C	
	5 min 95°C	x
Antigen retrieval	40 min or 25 min at 121°C	
**Step 4, Trypsin application**		
Trypsin concentration	1.3 ng/mm^2 (T2, HTX)	x
	3.3 ng/mm^2 (T1, SunCollect)	
	6.7 ng/mm^2 (T3, HTX)	
	16.7 ng/mm^2 (T4, SunCollect)	
**Step 5, Digestion**		
Trypsin digestion	2 hours at 50°C	
	17 hours at 37°C	x
Humidity chamber solvent	50% methanol	x
	Saturated K_2SO_4	x
**Step 6, Matrix application**		
CHCA matrix concentration	5 mg/mL	
	7 mg/mL	x
	10 mg/mL	
Matrix spray routine HTX sprayer	75°C, 10 layers, FR 0.060 mL/min, 7mg/mL, 1.8 µg/mm^2 (M1, HTX)	x
	75°C, 4 layers, FR 0.120 mL/min, 7mg/mL, 1.4 µg/mm^2 (M2, HTX)	
	75°C, 4 layers, FR 0.120 mL/min, 10mg/mL, 2.0 µg/mm^2 (M3, HTX)	x
	80°C, 6 layers, FR 0.10 mL/min, 7mg/mL, 1.6 µg/mm^2 (M4, HTX)	x
	Room temp., 10 layers, FR 0.37 mL/min, 0.7 µg/mm^2 (M5, SunCollect)	

Supplementary Table S3. Overview and ranking based on mean total QE score of all 25 methods including all parameters applied during sample preparation. QE = quality evaluation, n = number of sections and SD = standard deviation.

Rank	Method ID	Method parameters	n	mean total QE score ± SD
**1**	6	Tissue thickness: 10µm - Washing: ice-cold EtOH+H2O - Heating step: 5 min 95°C - Trypsin spraying: T2 - Incubate: 17h, 37°C - Matrix spraying: M1	9	6.26 ± 0.57
**2**	11	Tissue thickness: 10µm - Washing: RT EtOH+H2O - Heating step: AR - Trypsin spraying: T3 - Incubate: 17h, 37°C - Matrix spraying: M3	3	6.06 ± 0.23
**3**	5	Tissue thickness: 10µm - Washing: ice-cold EtOH+H2O - Heating step: 5 min 95°C - Trypsin spraying: T3 - Incubate: 17h, 37°C - Matrix spraying: M1	8	5.93 ± 0.35
**4**	9	Tissue thickness: 10µm - Washing: EtOH+short H2O - Heating step: AR - Trypsin spraying: T1 - Incubate: 17h, 37°C - Matrix spraying: M3	5	5.7 ± 0.18
**5**	25	Tissue thickness: 10µm - Washing: RT EtOH+H2O - Heating step: 5 min 95°C - Trypsin spraying: T2 - Incubate: 17h, 37°C - Matrix spraying: M4	3	5.5 ± 0.49
**6**	10	Tissue thickness: 10µm - Washing: RT EtOH+H2O - Heating step: 10 min 70C - Trypsin spraying: T2 - Incubate: 17h, 37°C - Matrix spraying: M1	6	5.4 ± 1.1
**7**	24	Tissue thickness: 10µm - Washing: ice-cold EtOH+H2O - Heating step: 5 min 95°C - Trypsin spraying: T2 - Incubate: 17h, 37°C - Matrix spraying: M4	6	5.4 ± 1.2
**8**	1	Tissue thickness: 10µm - Washing: RT EtOH+H2O - Heating step: AR - Trypsin spraying: T1 - Incubate: 17h, 37°C - Matrix spraying: M3	3	5.24 ± 0.12
**9**	20	Tissue thickness: 4µm - Washing: RT EtOH+H2O - Heating step: none - Trypsin spraying: T1 - Incubate: 2h, 50°C - Matrix spraying: M3	3	5.24 ± 0.17
**10**	12	Tissue thickness: 10µm - Washing: RT EtOH+H2O - Heating step: AR - Trypsin spraying: T1 - Incubate: 17h, 37°C - Matrix spraying: M5	5	4.9 ± 1.1
**11**	15	Tissue thickness: 10µm - Washing: RT EtOH+H2O - Heating step: none - Trypsin spraying: T2 - Incubate: 2h, 50°C - Matrix spraying: M2	10	4.73 ± 0.82
**12**	16	Tissue thickness: 10µm - Washing: RT EtOH+H2O - Heating step: none - Trypsin spraying: T2 - Incubate: 2h, 50°C - Matrix spraying: M4	5	4.48 ± 0.71
**13**	21	Tissue thickness: 4µm - Washing: RT EtOH+H2O - Heating step: AR - Trypsin spraying: T1 - Incubate: 17h, 37°C - Matrix spraying: M5	5	4.3 ± 0.59
**14**	14	Tissue thickness: 10µm - Washing: RT EtOH+H2O - Heating step: none - Trypsin spraying: T3 - Incubate: 17h, 37°C - Matrix spraying: M2	8	4.29 ± 0.48
**15**	2	Tissue thickness: 10µm - Washing: RT EtOH+H2O - Heating step: none - Trypsin spraying: T1 - Incubate: 2h, 50°C - Matrix spraying: M3	3	4.19 ± 0.31
**16**	4	Tissue thickness: 10µm - Washing: ice-cold EtOH+H2O - Heating step: 10 min 70C - Trypsin spraying: T2 - Incubate: 17h, 37°C - Matrix spraying: M1	8	3.96 ± 0.59
**17**	7	Tissue thickness: 10µm - Washing: ice-cold EtOH+H2O - Heating step: 5 min 95°C - Trypsin spraying: T2 - Incubate: 17h, 37°C - Matrix spraying: M1	4	3.93 ± 0.87
**18**	3	Tissue thickness: 10µm - Washing: ice-cold EtOH+H2O - Heating step: 10 min 70C - Trypsin spraying: T3 - Incubate: 17h, 37°C - Matrix spraying: M1	4	3.78 ± 0.92
**19**	23	Tissue thickness: 4µm - Washing: RT EtOH+H2O - Heating step: none - Trypsin spraying: T1 - Incubate: 2h, 50°C - Matrix spraying: M3	4	3.54 ± 0.7
**20**	17	Tissue thickness: 10µm - Washing: RT EtOH+H2O - Heating step: none - Trypsin spraying: T1 - Incubate: 2h, 50°C - Matrix spraying: M3	4	3.32 ± 0.65
**21**	19	Tissue thickness: 10µm - Washing: M4arnoy's - Heating step: AR - Trypsin spraying: T1 - Incubate: 17h, 37°C - Matrix spraying: M5	3	3.29 ± 0.31
**22**	18	Tissue thickness: 10µm - Washing: RT EtOH+H2O - Heating step: none - Trypsin spraying: T2 - Incubate: 17h, 37°C - Matrix spraying: M3	4	3.254 ± 0.079
**23**	22	Tissue thickness: 4µm - Washing: RT EtOH+H2O - Heating step: none - Trypsin spraying: T4 - Incubate: 2h, 50°C - Matrix spraying: M3	4	2.57 ± 0.41
**24**	8	Tissue thickness: 10µm - Washing: ice-cold EtOH+H2O - Heating step: none - Trypsin spraying: T2 - Incubate: 17h, 37°C - Matrix spraying: M1	4	2.51 ± 0.18
**25**	13	Tissue thickness: 10µm - Washing: RT EtOH+H2O - Heating step: none - Trypsin spraying: T4 - Incubate: 2h, 50°C - Matrix spraying: M3	4	2.47 ± 0.12
